# Enhancing the efficiency of high-order harmonics with two-color non-collinear wave mixing in silica

**DOI:** 10.1038/s41467-024-52774-9

**Published:** 2024-09-27

**Authors:** Sylvianne D. C. Roscam Abbing, Nataliia Kuzkova, Roy van der Linden, Filippo Campi, Brian de Keijzer, Corentin Morice, Zhuang-Yan Zhang, Maarten L. S. van der Geest, Peter M. Kraus

**Affiliations:** 1 Advanced Research Center for Nanolithography, Science Park 106, 1098 XG Amsterdam, The Netherlands; 2 Department of Physics and Astronomy, and LaserLaB, Vrije Universiteit, De Boelelaan 1105, 1081 HV Amsterdam, The Netherlands; 3 Institute for Theoretical Physics and Delta Institute for Theoretical Physics, University of Amsterdam, 1090 GL Amsterdam, The Netherlands

**Keywords:** Attosecond science, High-harmonic generation

## Abstract

The emission of high-order harmonics from solids under intense laser-pulse irradiation is revolutionizing our understanding of strong-field solid-light interactions, while simultaneously opening avenues towards novel, all-solid, coherent, short-wavelength table-top sources with tailored emission profiles and nanoscale light-field control. To date, broadband spectra in solids have been generated well into the extreme-ultraviolet (XUV), but the comparatively low conversion efficiency in the XUV range achieved under optimal conditions still lags behind gas-based high-harmonic generation (HHG) sources. Here, we demonstrate that two-color high-order harmonic wave mixing in a fused silica solid is more efficient than solid HHG driven by a single color. This finding has significant implications for compact XUV sources where gas-based HHG is not feasible, as solid XUV wave mixing surpasses solid-HHG in performance. Moreover, our results enable utilizing solid high-order harmonic wave mixing as a probe of structure or material dynamics of the generating solid, which will enable reducing measurement times compared to the less efficient regular solid HHG. The emission intensity scaling that follows perturbative optical wave mixing, combined with the angular separation of the emitted frequencies, makes our approach a decisive step for all-solid coherent XUV sources and for studying light-engineered materials.

## Abstract (web summary)

High-order harmonic generation in solids could develop into a compact, coherent, short-wavelength source. This study shows that two-colour noncollinear wave mixing in silica significantly enhances HHG efficiency over single-color methods, offering a novel pathway for advancing all-solid XUV sources.

## Introduction

High-harmonic generation (HHG) from gases has revolutionized ultrafast spectroscopy^1,2^, imaging science^3,4^, and is finding its way into first industrial applications^5–7^, but is still limited by the low conversion efficiency from infrared (IR) to extreme-ultraviolet (XUV) photons. A thorough understanding of the mechanism of gas-based HHG spurred the use of sculpted multi-color drivers that favorably modify the ionization rate, and thus increase the conversion efficiency^6,8–11^. High-harmonic generation from solids has emerged as a compelling area of research^12–17^, providing new possibilities for producing coherent XUV radiation^18–26^ while enabling the nanoscale engineering of solid-state materials to optimize their electronic properties and boost HHG efficiency^27,28^. The mechanisms of HHG in solids are still being debated. Different generation regimes exist and are characterized by the choice of the driving field and generation material. Proposed mechanisms include nonlinear intraband currents^12,14^, arising from carrier acceleration by the strong field after band-gap excitation, electron-hole recollisions after carrier excursion^13,19^ creating an interband polarization, ionization-induced injection currents^22^ for low-order harmonics, or treating emission as originating from dressed eigenstates in a strong static field, which under high excitation intensities drives Bloch oscillations^29^. The importance of carrier-injection dynamics in solid HHG^22,30^, combined with the observation of enhanced gas-based HHG for short-wavelength drivers, which favorably modifies the ionization step^31^ and thus enhances the conversion efficiency, points toward a new strategy: two-color noncollinear XUV generation, for both enhancing the efficiency of solid HHG, as well as shedding light on the generation mechanism.

## Results

### Observation of high-order wave mixing in silica

**Fig. 1:**
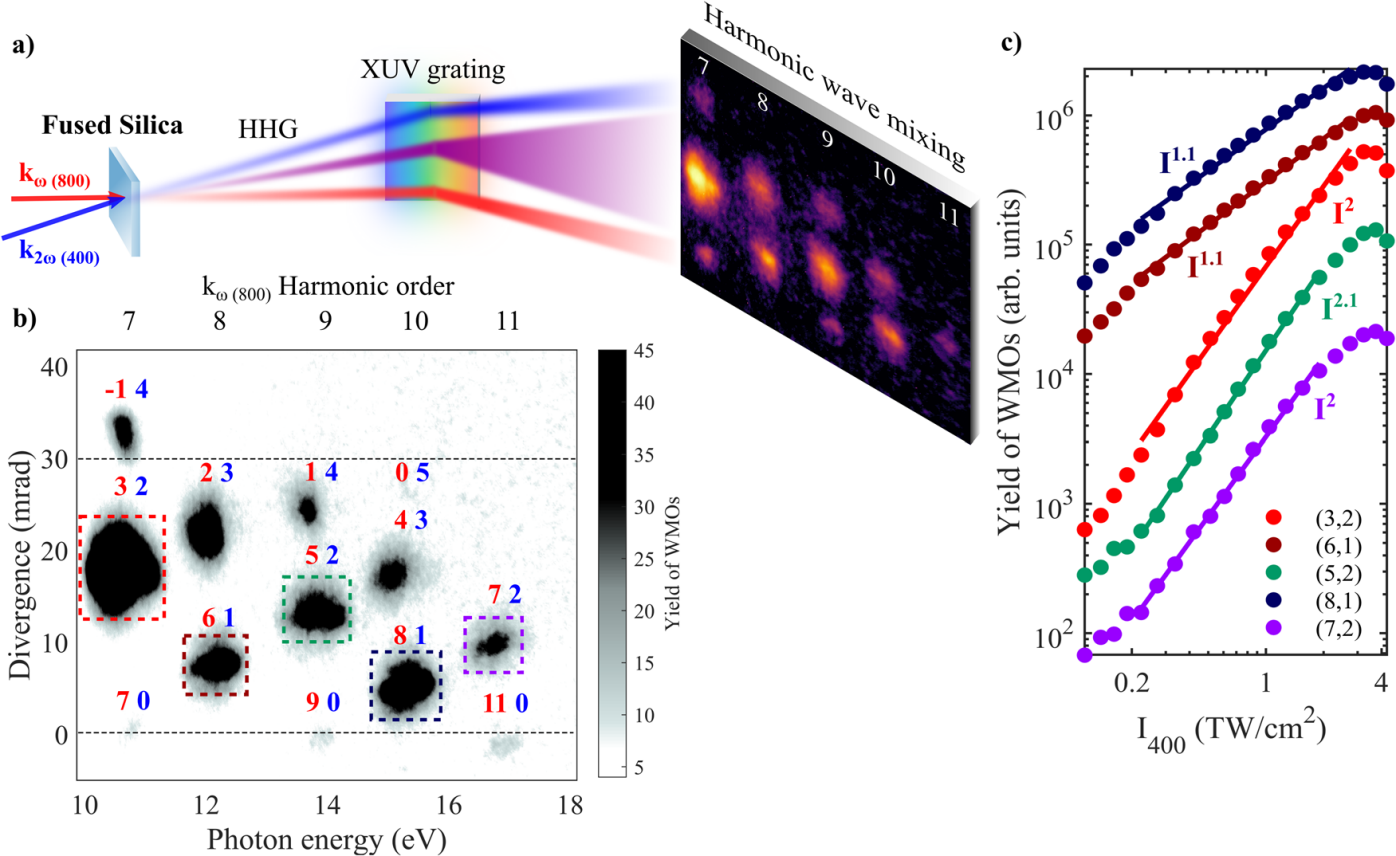
High-order wave mixing in fused silica. **a** Experimental arrangement. **b** Far-field spectrum of XUV wave mixing in silica. WMOs are spectrally dispersed horizontally and propagate vertically. WMOs generated only from 800 nm or 400 nm propagate at 0 mrad or 30 mrad, respectively. Harmonic orders refer to multiples of the 800-nm fundamental. **c** Yield of different WMOs as a function of the intensity of the 400-nm pulse. The solid lines are power laws, whose exponent is indicated in the plot. The different WMOs are vertically offset for clarity. The intensity of the 800-nm pulse was 10.5 TW/cm^2^.

Similar to non-collinear HHG in gases^32,33^, the angle between fundamental near-infrared (NIR, 800 nm) and second harmonic (SH, 400 nm) driving pulses in amorphous fused silica solid (SiO_2_) (Fig. 1a) provides an angular separation of the photon channels and prevents their overlap and interference, unlike in collinear two-color HHG^34^. Noncollinear HHG leads to an emission pattern (Fig. 1b) of harmonic wave mixing orders (WMOs) labeled (*n*, *m*), which follow momentum conservation and can thus be understood by wave vector addition of the underlying combination of *n* fundamental and *m* second harmonic photons. Only combinations of an odd total number of photons are dipole-allowed, due to the centrosymmetry of amorphous silica. More details are given in the supplementary information (SI), section I. The emission patterns in Fig. 1a, b show strongly enhanced WMOs compared to the angularly separated emission from the respective fundamental beams, which are indicated by horizontal dashed black lines in Fig. 1b. The separation of fundamental and SH photon channels in each WMO allows for identifying the effect of intensity scaling of the pulses on the harmonic emission, as shown in Fig. 1c for the scaling of the 400-nm pulse intensity (I_400_)—a first step in investigating and optimizing the photon-yield enhancement apparent in the WMOs. The yield of all WMOs faithfully follows a power-law trend over a large intensity range. The exponents of these power-law scalings (i.e., the slopes in a double logarithmic plot) are close to the number *m* of 400-nm photons involved, which would be the exponent for the scaling of the yield as a function of intensity in perturbative optical wave mixing.

### Simulations of wave mixing

**Fig. 2:**
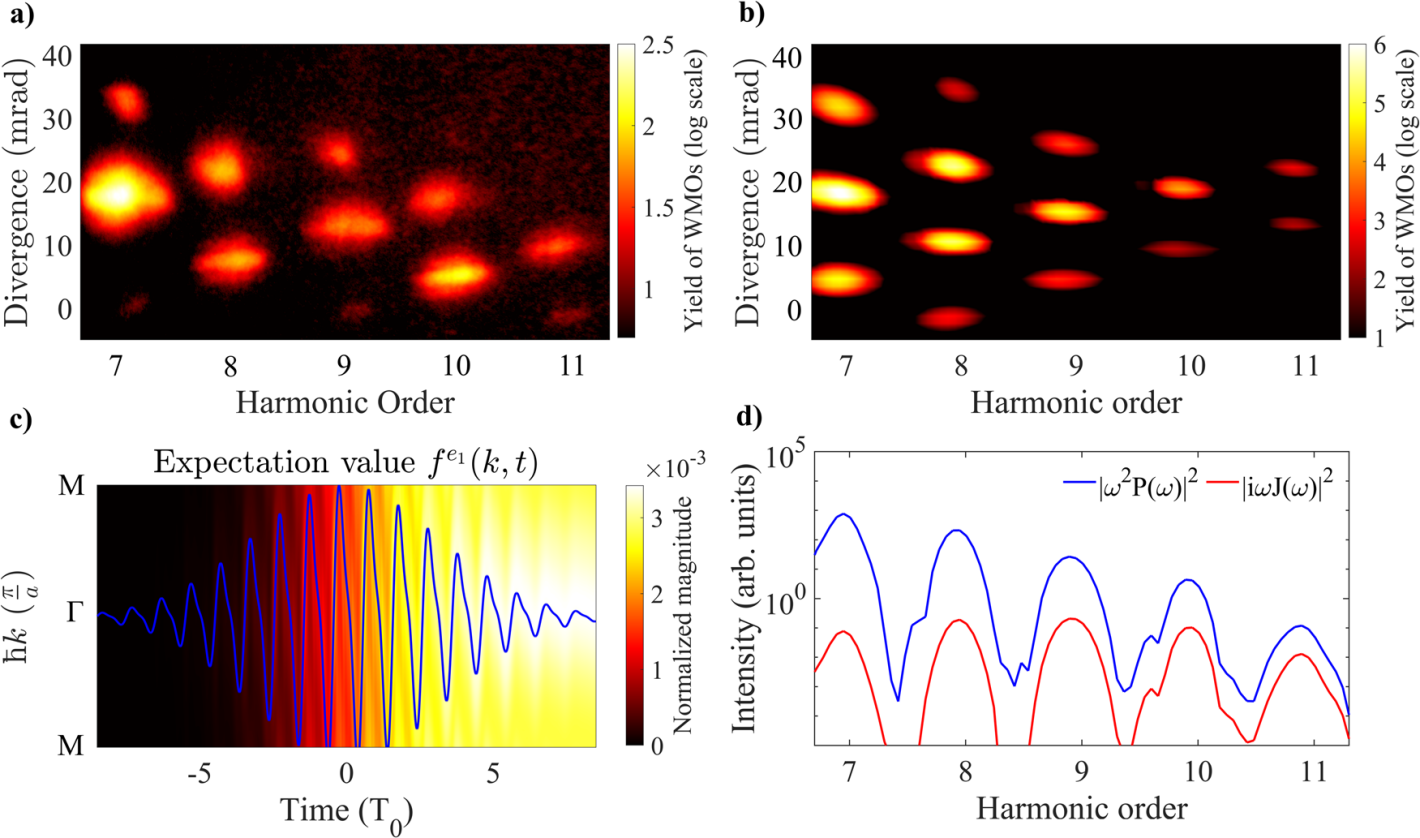
Simulation of solid-state high-harmonic generation. **a** Recorded far-field XUV wave mixing spectrum in silica. **b** Simulated far-field wave mixing spectrum using SBE calculations. **c** Simulated electron population ${f}^{{e}_{1}}(k,t)$ of the first conduction band along the *Γ*-M crystal direction, in momentum and time-space. The respective vector potential of the laser pulse is shown by the blue line. The cycle duration of the 800-nm pulse (2.7 fs) is denoted as *T*_0_. **d** Comparison between the contributions of the interband polarization (blue line) and intraband current (red line).

**Fig. 3:**
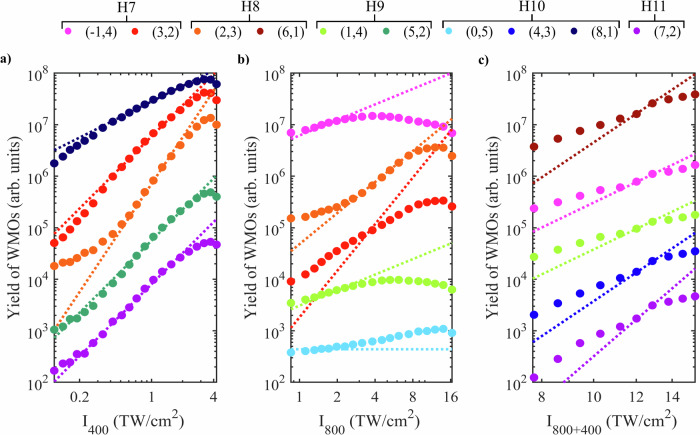
Experimental (circles) and semi-classically simulated (dashed lines) intensity scaling of different harmonic orders. **a** Only the intensity of the 400-nm pulse is scaled (the ratio I_400_/I_800_ varies between 0.01:1 and 0.4:1 from left to right on the horizontal intensity axis, I_800_ = 10.5 TW/cm^2^). **b** Only the intensity of the 800-nm pulse is scaled (the ratio I_800_/I_400_ varies between 0.5:1 to 10.3:1 from left to right on the horizontal intensity axis, I_400_ = 1.6 TW/cm^2^). **c** The total intensity of two colors is scaled (the ratio I_800_/I_400_ is 3.3:1). For better visualization of the WMOs, the curves are vertically shifted. Harmonic orders (H7-11) refer to multiples of the 800-nm fundamental. The color coding is consistent across the panels.

In order to understand the origin of the enhanced WMOs (Fig. 1b) and the perturbative intensity scalings (Fig. 1c), we solve the semiconductor Bloch equations (SBE)^35–37^ to simulate solid-state harmonic generation. The field-driven carrier population exchange between the bands leads to the generation of an interband polarization and an intraband current (SI, section II A). This model was solved for a grid of points, which was spanned by the crossed 800-nm and 400-nm pulses that form a spatial interference grating. The resulting spatially modulated XUV emission was then propagated to the far field by Fraunhofer diffraction (Fig. 2b), resulting in an excellent reproduction of the experimental wave mixing pattern (Fig. 2a). Zooming into the attosecond electron dynamics in k-space that drive HHG, we observe that the intense two-color laser field excites carriers from the valence band to the first conduction band not just at the *Γ*-point but all across *k*-space. Simultaneously, the field accelerates the carriers along the energy bands, resulting in carrier trajectories in *k*-space that trace out the vector potential of the field (Fig. 2c). Due to the high ponderomotive energy the carriers are accelerated beyond the first edge of the Brillouin zone and undergo Bloch oscillations. The concentration of carriers at a given *k*-point undergoes rapid changes in time, inducing a high-frequency interband polarization that radiates light at high photon energies, as shown in Fig. 2d. The radiation due to the carrier acceleration inside the bands, the intraband current, is significantly lower than the radiation from interband polarization for the harmonic energies measured in this manuscript. Both the interband and intraband mechanisms have in common that they are induced by the laser-driven motion of carriers inside the bands. To identify if this laser-driven carrier motion gives rise to the perturbative intensity scalings, we model the harmonic emission originating only from the electron group velocity, which we describe semi-classically^14,38^ using a time-dependent crystal momentum which traces out the shape of the band during acceleration (SI, section II E). We use this numerical approach for simulating perturbative wave mixing and show the results in Fig. 3.

### Intensity scaling

A detailed investigation of the experimental (circles) and semi-classically simulated (dashed lines) yields of the WMOs as a function of the intensity of the driving fields is shown in Fig. 3 for selected representative wave mixing orders and in section III of the SI for the remaining wave mixing orders. Figure 3a shows an intensity scaling of the 400-nm pulses, I_400_, from 0.1 TW/cm^2^ to 4.2 TW/cm^2^ wherein the intensity of the fundamental 800-nm field is kept constant, I_800_ = 10.5 TW/cm^2^. The dashed lines in Fig. 3a show that the simulated trend of a specific harmonic order (*n*, *m*) reproduces the highly nonlinear dependence of the scaled driving field. We observe saturation of the XUV yield at high intensities before approaching the damage threshold of the sample. Next, experiments and simulations were performed for the constant intensity of the 400-nm pulse, I_400_ = 1.6 TW/cm^2^, while the intensity of the 800-nm pulse, I_800_, was increased in steps from 0.85 TW/cm^2^ to 16.4 TW/cm^2^, as shown in Fig. 3b. The behavior in both the experiment and simulations resembles that of a perturbative process. WMOs that contain a higher portion of 800-nm photons deviate from a pure power law and rise less steeply, as observed for WMO (3,2), therefore deviating from a pure perturbative scaling and the (perturbative) simulation. Figure 3c shows a scaling of the total intensity, I_400+800_, where the energy of the two pulses is increased independently in steps, to keep the ratio of intensities I_800_/I_400_ = 3.3:1 constant across the entire intensity range. Most wave mixing orders follow a power law of below 5 for these 5th-order processes, whereas the simulations catch the perturbative behavior. As expected, a total power intensity scaling is less clearly described as a perturbative process. Overall, the generally good agreement between experiments and simulations for the 400-nm intensity scans in Fig. 3a and the fair (but more deviating) scalings for 800-nm and total intensity in Fig. 3b, c confirm that the laser-driven carrier motion drives the XUV generation of solid-state HHG, resulting in a perturbative scaling for large intensity ranges. This has implications for XUV sources based on solid HHG. The perturbative response causes the yield of solid HHG as a function of driving intensity to rise with an exponent determined by the number of photons (*n*, *m*) involved in HHG. This enables a selective enhancement of an individual or a selection of WMOs, depending on the relative strength of the two driving fields. Together with the natural angular separation of WMOs, this illustrates that XUV wave mixing in solids can help to realize an all-solid single-element XUV generator and multi-wavelength beamsplitter, where different energy emissions are mapped onto different emission angles. Further separation of the remaining energies in a beam could be realized by the simple addition of a hard aperture, without the need for lossy metal filters, or reducing the number or thickness of filters. In addition, we can infer from the experimental wave mixing patterns that there is a significant increase in harmonic yield for WMOs compared to single-color harmonics.

### Enhancing the efficiency of XUV generation via wave mixing

In Fig. 2a, WMO (3,2) exhibits the strongest emission, while WMO (5,2) is the most intense at 14 eV photon energy, and the most intense emission at 17 eV is from WMO (7,2). Through a comparison of the yields of on-axis and WM harmonic orders at the same photon energy using single or two-color drivers, it was observed that the XUV wave mixing yield of WMOs (3,2) and (5,2) was enhanced by almost two orders of magnitude compared to harmonic orders (7,0) and (9,0) that occur at the same photon energy when driven solely by an 800-nm pulse (SI, section III B). To quantify the increase in WM yield in silica, we conducted separate measurements implementing photon flux detection using xenon gas and compared the spectral amplitude of recorded far-field WM patterns in silica with those of xenon (see details in SI, section IV). Therein, the total conversion efficiency for WM in silica was determined to be 5.60 ± 1.16 × 10^−10^ (2.77 ±  0.59 × 10^7^ photons/second at 1 kHz repetition rate), while the efficiency for 800-nm single-color solid HHG over the same 10–13 eV photon energy range was found to be one to two orders of magnitude lower, depending on the harmonic order. Hence, our results validate that the two-color WM conversion efficiency in silica surpasses that of NIR single-color solid HHG by a factor of at least ten. Previously reported single-color solid HHG photon flux values were 10^9^–10^10^ photons/second^39^, which, to the best of our knowledge, represents the only reported measurement of XUV HHG from solids. Luu and Wörner^39^ operated at a higher average power (due to a higher repetition rate of the driver) and used different (quartz) and thinner (20 μm) materials, whereas our sample was a 100 μm-thick (amorphous) fused silica. Moreover, the shorter wavelength and lower repetition rate of the NIR driver laser system used in this work, which differs from those in previous research^39^, can have a considerable impact on the efficiency of high-harmonic emissions. Importantly, our findings clearly demonstrate that the XUV yield increases significantly—by nearly two orders of magnitude—in the two-color wave mixing scenario.

## Discussion

**Fig. 4:**
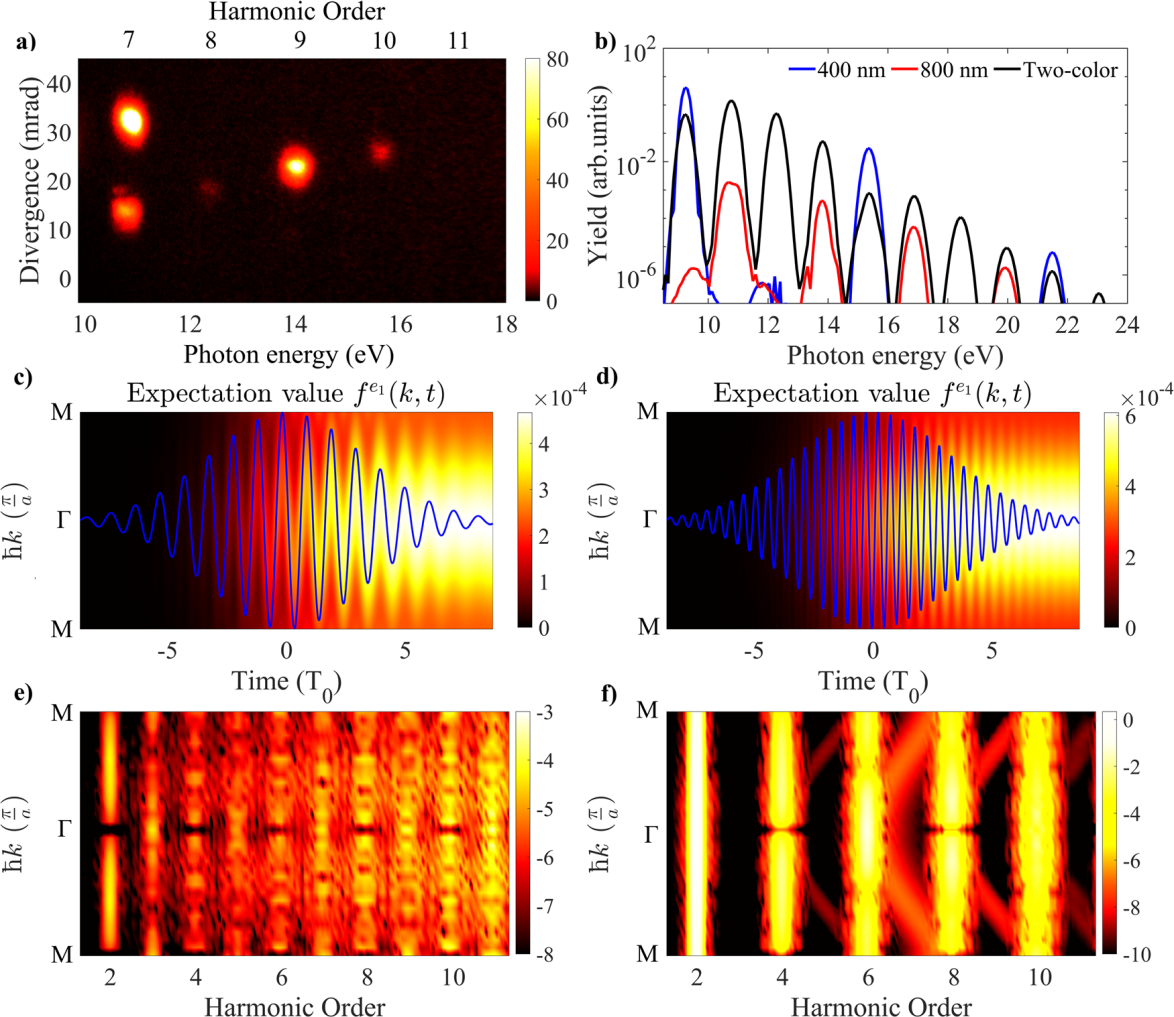
Comparison of 800-nm and 400-nm driven solid-state high-harmonic generation. **a** Experimentally measured far-field wave mixing spectrum in silica with equal 400-nm and 800-nm intensities of 1.6 TW/cm^2^. **b** Simulated spectra, comparing 400-nm, 800-nm, and two-color driving pulses with equal intensity. **c** Electrons in the first conduction band as a function of time and crystal momentum for an 800-nm pulse (vector potential represented by the blue line), and **d** for a 400-nm pulse. **e** Energy- and momentum-resolved polarization for 800 nm, and **f** for 400 nm, both for an intensity of 1.6 TW/cm^2^. The color bar represents harmonic yield on a logarithmic scale. Harmonic orders refer to multiples of the 800-nm fundamental, with even orders of the driving frequencies canceling out in the emission spectrum due to symmetry.

We now delve deeper into the investigation of what enhances the efficiency of WMOs, we first experimentally verify the relative importance of both driving wavelengths. When we generate with both the fundamental and SH field at equal intensities (each 1.6 TW/cm^2^), we observe that the predominant harmonic emission occurs within WMOs that are along the 400-nm divergence direction as depicted in Fig. 4a, indicating a primary contribution of the 400-nm field to the WM pattern. Simulations involving equal intensities of 800-nm, 400-nm, or two-color pulses in Fig. 4b indeed confirm that enhancing HHG by aiding a shorter wavelength leads to the highest harmonic emission. Both in experiment and theory, the appropriate delay between the two-color pulses was chosen such that the envelopes of the pulses overlapped to generate the most efficient wave mixing signal. When comparing the momentum and time-resolved photocarrier excitation for 800-nm and 400-nm pulses in Fig. 4c, d, two key observations are made: First, the use of a short driving wavelength increases the ionization probability and thus the number of excited carriers. Second, the shorter driving wavelength accelerates carriers across *k* with a higher frequency, leading to an enhanced carrier concentration around the *Γ*-point, where the band curvature facilitates both large carrier velocity and acceleration. This combination of increased photoexcitation and high-frequency light-induced carrier motion results in a larger polarization for shorter driving wavelengths. This effect ultimately enhances high-order harmonic emission in the XUV, as evident from the amplitude for the energy- and momentum-resolved time-integrated polarization (Fig. 4e, f), which is higher for a 400-nm driver (Fig. 4f) than for an 800-nm driver (Fig. 4e). Mixing the two driving fields of 800 nm and 400 nm additionally results in the advantageous combination of high yield and broad spectral coverage. The driving force of enhanced carrier concentration is most prominently seen through the *k*-resolved and energy-resolved polarizations in Fig. 4e, f. When driving HHG with 400 nm (Fig. 4f), replicas of the conduction band (curved, weaker signals) at multiples of the driving photon energy are clearly visible. These laser-dressed states—the Floquet–Bloch states^40,41^ - have recently received a lot of attention as they are directly observable in photoelectron spectroscopy. Here, we interpret the enhanced emission efficiency in high-order wave mixing as transitions between such efficiently formed Floquet–Bloch states that give rise to the polarization signals at energies corresponding to multiples of the *k*-dependent band energies, as visible by the straight and intense emission signals in Fig. 4f. This illustrates that the shorter driving field operates further in the multiphoton regime providing assistance to more efficient carrier excitation and thus overall boosting HHG emission intensity. We note that the curved signal in Fig. 4f, which directly follows the population of Floquet–Bloch states, in principle indicates weak emission at wavelengths that correspond to dressing of the bandgap. We did not observe such emission, and we would not expect it, as the dephasing of such states is too fast^41^ to lead to any sufficiently narrow emission that we could observe.

In conclusion, we have demonstrated that the efficiency of high-order harmonics can be enhanced by utilizing two-color non-collinear wave mixing in silica. Our theory based on semiconductor Bloch equations revealed that both Floquet–Bloch dressing followed by interband and intraband dynamics compete during intense laser-irradiation promoting high-efficiency carrier excitation and thus boosting the HHG emission intensity. The resulting brightness enhancement turns solid HHG into a competitive source, especially in combination with the recently pioneered wavefront shaping of solid HHG through nanostructures^28^. Moreover, our simulations show that harmonics can be understood as signatures of laser dressing and Floquet engineering of materials^42,43^, a topic that has recently gathered much attention due to the opportunity to modify electronic structures of solids at will purely through light-matter interaction^44,45^. In addition, the multiband dynamics that drive wave mixing in this study correspond to a field-driven attosecond electron wave packet motion in the crystal lattice. The simultaneous recording of multiple experimental observables from solid HHG may thus allow for the reconstruction of this attosecond electron motion as previously achieved in molecules^46^, and become a valuable tool for investigating laser-driven phase transitions and strongly correlated electron dynamics through high-harmonic spectroscopy^47^, with the possibility to add picometer spatial resolution as demonstrated for solid HHG recently^48^.

## Methods

### Sample preparation

The double-side polished, 100 μm-thick amorphous fused silica sample (purchased from UQG Optics) was utilized, which was pre-cleaned with a standard cleaning procedure (H_2_O/30% HCl/30% H_2_O_2_ in a 5:1:1 ratio) at 70 °C for 15 min, followed by isopropanol cleaning, prior to the experiments.

### Experimental setup

The part of the output of a Ti: sapphire laser amplifier (Coherent Astrella, 800 nm, 4 mJ, 50 fs, 1 kHz) was split into two arms. In one arm, 400-nm pulses were generated via second harmonic generation in a 200 μm-thick *β*-barium borate (BBO) crystal. In the second arm, with 800-nm pulses, a delay line was implemented to control the temporal overlap of the two pulses onto the sample. Half-wave plates were placed in both arms to ensure parallel polarization of the two beams. The 400 and 800-nm beam intensities were further attenuated by neutral density filters placed in two arms before focusing on the sample to control the peak intensities and to prevent solid damage. Focusing was achieved using a spherical mirror with a focal length of 750 mm. The crossing angle of 30 mrad in focus was implemented by impinging onto the spherical mirror with the two beams parallel and displaced in height by 9 mm. For a better spatial overlap, the focal spot sizes of the 400 and 800-nm beams were chosen to be approximately the same (83 μm and 108 μm (1/e^2^), respectively). The peak intensity for each driving pulse with a Gaussian envelope was determined in vacuum as I = 1.88  × (E/(*τ*  ⋅ (*π*  ⋅ ${\omega }_{0}^{2}$)), where E is the pulse energy, *τ* is the pulse duration, and *ω*_0_ is the focal spot size diameter defined at 1/e^2^. Considering also the reflection loss from the boundary between the silica sample and vacuum, the experimental peak intensity was calculated as I_*λ*_  = I × 4n_*λ*_/(n_*λ*_ + 1)^2^, where *λ* is the driving 400 or 800 nm wavelength, and n is the wavelength-dependent refractive index of SiO_2_ material (n_400_ = 1.4701 and n_800_ = 1.4533). After the sample, the generated harmonics were spectrally dispersed along the horizontal direction with an aberration-corrected, concave, flat-field grating (1200 lines/mm) and detected by a double-stack micro-channel plate (MCP) assembly, backed with a phosphor screen, which was imaged with a CMOS camera from outside the vacuum chamber.

### Simulations

We model HHG from fused silica as a strongly driven system of one valence band and two conduction bands for a 40-fs pulse along the *Γ*-M crystal direction. The energies and the strength of the dipole coupling are calculated within density functional theory, using pseudopotentials and a plane-wave basis set as implemented in Quantum Espresso. The high-harmonic emission is calculated by solving the semiconductor Bloch equations. The far-field pattern is obtained by propagating the emission pattern to the far field by Fraunhofer diffraction. Details are given in the SI, section II.

## Supplementary information

Supplementary Information

## Data Availability

The data that support the plots within this paper and other findings of this study are available from P.M.K. upon request.

## Code availability

The codes that support the findings of this study are available from P.M.K. upon request.

## References

[1] Kraus, P. M., Zürch, M., Cushing, S. K., Neumark, D. M. & Leone, S. R. The ultrafast X-ray spectroscopic revolution in chemical dynamics. *Nat. Rev. Chem.* **2**, 82–94 (2018).

[2] Kraus, P. M. & Wörner, H. J. Perspectives of attosecond spectroscopy for the understanding of fundamental electron correlations. *Angew. Chem. Int. Ed.* **57**, 5228–5247 (2018). 10.1002/anie.201702759 29624808

[3] Miao, J., Ishikawa, T., Robinson, I. K. & Murnane, M. M. Beyond crystallography: diffractive imaging using coherent X-ray light sources. *Science* **348**, 530–535 (2015). 25931551 10.1126/science.aaa1394

[4] Witte, S., Tenner, V. T., Noom, D. W. & Eikema, K. S. Lensless diffractive imaging with ultra-broadband table-top sources: from infrared to extreme-ultraviolet wavelengths. *Light Sci. Appl.* **3**, e163 (2014).

[5] Ku, Y.-S. et al. EUV scatterometer with a high-harmonic-generation EUV source. *Opt. Express* **24**, 28014–28025 (2016). 27906368 10.1364/OE.24.028014

[6] Roscam Abbing, S. et al. Divergence control of high-harmonic generation. *Phys. Rev. Appl.* **13**, 054029 (2020).

[7] Roscam Abbing, S. D. C., Campi, F., Zeltsi, A., Smorenburg, P. & Kraus, P. M. Divergence and efficiency optimization in polarization-controlled two-color high-harmonic generation. *Sci. Rep.* **11**, 24253 (2021). 34930994 10.1038/s41598-021-03657-2 PMC8688547

[8] Kim, I. J. et al. Highly efficient high-harmonic generation in an orthogonally polarized two-color laser field. *Phys. Rev. Lett.* **94**, 243901 (2005).

[9] Brizuela, F. et al. Efficient high-order harmonic generation boosted by below-threshold harmonics. *Sci. Rep.* **3**, 1–5 (2013). 10.1038/srep01410 PMC3593216 23475106

[10] Haessler, S. et al. Optimization of quantum trajectories driven by strong-field waveforms. *Phys. Rev. X* **4**, 021028 (2014).

[11] Rajeev, R. et al. In situ frequency gating and beam splitting of vacuum- and extreme-ultraviolet pulses. *Light Sci. Appl*. **5**, e16170-e16170 (2016). 10.1038/lsa.2016.170 PMC6059825 30167130

[12] Ghimire, S. et al. Observation of high-order harmonic generation in a bulk crystal. *Nat. Phys.* **7**, 138–141 (2011).

[13] Schubert, O. et al. Sub-cycle control of terahertz high-harmonic generation by dynamical Bloch oscillations. *Nat. Photonics* **8**, 119–123 (2014).

[14] Luu, T. T. et al. Extreme ultraviolet high-harmonic spectroscopy of solids. *Nature* **521**, 498–502 (2015). 26017451 10.1038/nature14456

[15] Yong Sing, Y. et al. High-harmonic generation in amorphous solids. *Nat. Commun.* **8**, 724 (2017). 28959029 10.1038/s41467-017-00989-4 PMC5620047

[16] Golde, D., Meier, T. & Koch, S. W. High harmonics generated in semiconductor nanostructures by the coupled dynamics of optical inter- and intraband excitations. *Phys. Rev. B* **77**, 075330 (2008).

[17] Ghimire, S. & Reis, D. High-harmonic generation from solids. *Nat. Phys.* **15**, 10–16 (2019).

[18] Luu, T. T., Scagnoli, V., Saha, S., Heyderman, L. J. & Wörner, H. J. Generation of coherent extreme ultraviolet radiation from *α* - quartz using 50 fs laser pulses at a 1030 nm wavelength and high repetition rates. *Opt. Lett.* **43**, 1790–1793 (2018). 29652365 10.1364/OL.43.001790

[19] Vampa, G. et al. Linking high harmonics from gases and solids. *Nature* **522**, 462–464 (2015). 26108855 10.1038/nature14517

[20] Yoshikawa, N., Tamaya, T. & Tanaka, K. High-harmonic generation in graphene enhanced by elliptically polarized light excitation. *Science* **356**, 736–738 (2017). 28522530 10.1126/science.aam8861

[21] Hafez, H. A. et al. Extremely efficient terahertz high-harmonic generation in graphene by hot Dirac fermions. *Nature* **561**, 507–511 (2018). 30202091 10.1038/s41586-018-0508-1

[22] Jürgens, P. et al. Origin of strong-field-induced low-order harmonic generation in amorphous quartz. *Nat. Phys.* **16**, 1035–1039 (2020).

[23] Ndabashimiye, G. et al. Solid-state harmonics beyond the atomic limit. *Nature* **534**, 520–523 (2016). 27281195 10.1038/nature17660

[24] Li, J. et al. Attosecond science based on high harmonic generation from gases and solids. *Nat. Commun.* **11**, 2748 (2020). 32488005 10.1038/s41467-020-16480-6 PMC7265550

[25] Han, S. et al. Extraction of higher-order nonlinear electronic response in solids using high harmonic generation. *Nat. Commun.* **10**, 3272 (2019). 31332192 10.1038/s41467-019-11096-x PMC6646338

[26] Uzan, A. J. et al. Attosecond spectral singularities in solid-state high-harmonic generation. *Nat. Photonics* **14**, 183–187 (2020).

[27] Franz, D. et al. All semiconductor enhanced high-harmonic generation from a single nanostructured cone. *Sci. Rep.* **9**, 5663 (2019). 30952870 10.1038/s41598-019-41642-y PMC6450872

[28] Roscam Abbing, S. D. C. et al. Extreme-ultraviolet shaping and imaging by high-harmonic generation from nanostructured silica. *Phys. Rev. Lett.* **128**, 223902 (2022). 35714263 10.1103/PhysRevLett.128.223902

[29] Higuchi, T., Stockman, M. I. & Hommelhoff, P. Strong-field perspective on high-harmonic radiation from bulk solids. *Phys. Rev. Lett.* **113**, 213901 (2014). 25479494 10.1103/PhysRevLett.113.213901

[30] Lang, Y., Peng, Z., Liu, J., Zhao, Z. & Ghimire, S. Proposal for high-energy cutoff extension of optical harmonics of solid materials using the example of a one-dimensional ZnO crystal. *Phys. Rev. Lett.* **129**, 167402 (2022). 36306748 10.1103/PhysRevLett.129.167402

[31] Lai, C.-J. et al. Wavelength scaling of high harmonic generation close to the multiphoton ionization regime. *Phys. Rev. Lett.* **111**, 073901 (2013). 23992066 10.1103/PhysRevLett.111.073901

[32] Bertrand, J. B. et al. Ultrahigh-order wave mixing in noncollinear high harmonic generation. *Phys. Rev. Lett.* **106**, 023001 (2011). 21405226 10.1103/PhysRevLett.106.023001

[33] Heyl, C. M. et al. Macroscopic effects in noncollinear high-order harmonic generation. *Phys. Rev. Lett.* **112**, 143902 (2014). 24765964 10.1103/PhysRevLett.112.143902

[34] Li, L. et al. Scaling law of high harmonic generation in the framework of photon channels. *Phys. Rev. Lett.* **120**, 223203 (2018). 29906171 10.1103/PhysRevLett.120.223203

[35] Lindberg, M. & Koch, S. W. Effective bloch equations for semiconductors. *Phys. Rev. B* **38**, 3342–3350 (1988). 10.1103/physrevb.38.3342 9946675

[36] Yue, L. & Gaarde, M. B. Introduction to theory of high-harmonic generation in solids: tutorial. *J. Opt. Soc. Am. B.* **39**, 535–555 (2022).

[37] Baykusheva, D. et al. Strong-field physics in three-dimensional topological insulators. *Phys. Rev. A* **103**, 023101 (2021).

[38] Wegener, M. *Extreme Nonlinear Optics: An Introduction* (Springer Berlin, Heidelberg, 2005).

[39] Luu, T. T. & Wörner, H. J. Measurement of the Berry curvature of solids using high-harmonic spectroscopy. *Nat. Commun.* **9**, 916 (2018). 29500349 10.1038/s41467-018-03397-4 PMC5834542

[40] Wang, Y. H., Steinberg, H., Jarillo-Herrero, P. & Gedik, N. Observation of Floquet-Bloch states on the surface of a topological insulator. *Science* **342**, 453–457 (2013). 24159040 10.1126/science.1239834

[41] Ito, S. et al. Build-up and dephasing of Floquet-Bloch bands on subcycle timescales. *Nature* **616**, 696–701 (2023). 37046087 10.1038/s41586-023-05850-x

[42] Perez-Piskunow, P. M., Usaj, G., Balseiro, C. A. & Torres, L. E. F. F. Floquet chiral edge states in graphene. *Phys. Rev. B* **89**, 121401 (2014).

[43] Oka, T. & Kitamura, S. Floquet engineering of quantum materials. *Annu. Rev. Condens. Matter Phys.* **10**, 387–408 (2019).

[44] Lindner, N. H., Refael, G. & Galitski, V. Floquet topological insulator in semiconductor quantum wells. *Nat. Phys.* **7**, 490–495 (2011).

[45] McIver, J. W. et al. Light-induced anomalous Hall effect in graphene. *Nat. Phys.* **16**, 38–41 (2020). 31915458 10.1038/s41567-019-0698-y PMC6949120

[46] Kraus, P. M. et al. Measurement and laser control of attosecond charge migration in ionized iodoacetylene. *Science* **350**, 790–795 (2015). 26494175 10.1126/science.aab2160

[47] Silva, R. E. F., Blinov, I. V., Rubtsov, A. N., Smirnova, O. & Ivanov, M. High-harmonic spectroscopy of ultrafast many-body dynamics in strongly correlated systems. *Nat. Photonics* **12**, 266–270 (2018).

[48] Lakhotia, H. et al. Laser picoscopy of valence electrons in solids. *Nature* **583**, 55–59 (2020). 32612227 10.1038/s41586-020-2429-z

