# Supplementary material for: Enhancing the efficiency of high-order harmonics with two-color non-collinear wave mixing in silica
Source: Nat Commun. 2024 Sep 27;15:8335. doi: 10.1038/s41467-024-52774-9 (PMC11436833; doi:10.1038/s41467-024-52774-9)
Supplement: Supplementary file 1 — Supplementary Information [file 41467_2024_52774_MOESM1_ESM.pdf]

Supplementary Information: Enhancing the efficiency of high-order harmonics with two-color non-collinear wave mixing in silica

Sylvianne D.C. Roscam Abbing[1], Nataliia Kuzkova[1,3], Roy van der Linden[1], Filippo Campi[1], Brian de Keijzer[1], Corentin Morice[2], Zhuang-Yan Zhang[1], Maarten L.S. van der Geest[1], and Peter M. Kraus[1,3]*

1 Advanced Research Center for Nanolithography, Science Park 106, 1098 XG Amsterdam, The Netherlands
2 Institute for Theoretical Physics Amsterdam and Delta Institute for Theoretical Physics, University of Amsterdam, 1098 XH Amsterdam, Netherlands
3 Department of Physics and Astronomy, and LaserLaB, Vrije Universiteit, De Boelelaan 1081, 1081 HV Amsterdam, The Netherlands

(Dated: September 10, 2024)

I. SELECTION RULES AND EMISSION ANGLE FOR HIGH-ORDER WAVE MIXING

Three selection rules must be considered to understand non-collinear wave mixing spectra driven by the fundamental and its second harmonic, and to assign each harmonic order to a unique combination of photons. Both wavelengths contribute to the emitted photon energies via an integer number of photons. From a simple energy conservation standpoint, the expected emission takes place at photon energies: $q\omega_f = n\omega_f + m2\omega_f = (n+2m)\omega_f$, where $q = n+2m$ is the harmonic order, ω_f is the angular frequency of the fundamental laser field, and n and m are the numbers of photons from the fundamental and its second harmonic, respectively. This yields a discrete spectrum, shown in Fig. 1b. Emission corresponding to even harmonics of the fundamental is present, despite the fact that fused silica is a centrosymmetric material. In fact, in a two-color field of commensurate frequencies, the parity conservation rule, which requires the total number of photons $n + m =$ to be odd, allows for generation of even harmonics of the fundamental through the addition (subtraction) of an odd amount of photons. The third conservation rule needed for a description of the full far-field profile shown in Fig. 1b, is conservation of the momentum. The harmonic spectrum spans less then an octave in energy, resulting into a unique mapping of each wave-mixing order (WMO) onto its emission angle β for a crossing angle α between fundamental and the second harmonic,

$$\beta(n,m) = \frac{2m \sin\alpha}{n + 2m\cos\alpha}. \tag{1}$$

A small-angle approximation is used in Eq. 1. Similar concepts have previously been applied in gas-phase HHG [1, 2].

II. THEORY OF HIGH-ORDER WAVE MIXING SPECTRA

A. Semiconductor Bloch equations

We model the generation of high-harmonics from silica, by solving the semiconductor Bloch equations [3–5] for a three-level system, consisting of one valance band and two conduction bands. Throughout this manuscript we use atomic units. The system of coupled differential equations are solved for the momentum k dependent population f_k^λ in band λ and the polarization $p_k^{\lambda\lambda'}$ between bands λ and λ', and is written out for bands $\lambda \in \{h_1, e_1, e_2\}$ as:

$$i\frac{\partial}{\partial t}p_k^{h_1e_1} = (\epsilon_k^{e_1} + \epsilon_k^{h_1} - i\frac{1}{T_2})p_k^{h_1e_1} - (1 - f_k^{e_1} - f_k^{h_1})d_k^{e_1h_1}F(t) + iF(t)\cdot\nabla_k p_k^{h_1e_1} + F(t)[d_k^{e_2h_1}p_k^{e_2e_1} - d_k^{e_1e_2}p_k^{h_1e_2}] \tag{2}$$

$$i\frac{\partial}{\partial t}p_k^{h_1e_2} = (\epsilon_k^{e_2} + \epsilon_k^{h_1} - i\frac{1}{T_2})p_k^{h_1e_2} - (1 - f_k^{e_2} - f_k^{h_1})d_k^{e_2h_1}F(t) + iF(t)\cdot\nabla_k p_k^{h_1e_2} + F(t)[d_k^{e_1h_1}p_k^{e_2e_1} - d_k^{e_1e_2}p_k^{h_1e_1}] \tag{3}$$

---

*Electronic address: p.kraus@arcnl.nl

$$i\frac{\partial}{\partial t}p_k^{e_1e_2} = (\epsilon_k^{e_2} - \epsilon_k^{e_1} - i\frac{1}{T_2})p_k^{e_1e_2} + (f_k^{e_2} - f_k^{e_1})d_k^{e_2e_1}F(t) + iF(t)\cdot\nabla_k p_k^{e_1e_2} + F(t)[d_k^{e_1h_1}p_k^{e_2h_1} - d_k^{h_1e_2}(p_k^{h_1e_1})^*] \tag{4}$$

$$\frac{\partial}{\partial t}f_k^{e_1} = -2\ \mathrm{Im}[d_k^{e_1e_2}F(t)(p_k^{e_2e_1})^* + d_k^{e_1h_1}F(t)(p_k^{h_1e_1})^*] + F(t)\cdot\nabla_k f_k^{e_1}. \tag{5}$$

$$\frac{\partial}{\partial t}f_k^{e_2} = -2\ \mathrm{Im}[d_k^{e_2e_1}F(t)(p_k^{e_1e_2})^* + d_k^{e_2h_1}F(t)(p_k^{h_1e_2})^*] + F(t)\cdot\nabla_k f_k^{e_2}. \tag{6}$$

$$\frac{\partial}{\partial t}f_k^{h_1} = -2\ \mathrm{Im}[d_k^{e_1h_1}F(t)(p_k^{h_1e_1})^* + d_k^{e_2h_1}F(t)(p_k^{h_1e_2})^*] + F(t)\cdot\nabla_k f_k^{h_1}. \tag{7}$$

in which, the single particle energies of the carriers in band λ are given by ϵ_k^λ. The creation of polarization and population due to the presence of an electric field $F(t)$ follows from the terms that involve the transition dipole moment $d_k^{\lambda\lambda'}$. The intraband dynamics, caused by carrier acceleration by $F(t)$ within the bands, are described by the terms including ∇_k. The dephasing time of the polarization is denoted by T_2. The transition dipole moment $d_k^{\lambda\lambda'}$ between the bands λ and λ' is approximated in first order $\mathbf{k}\cdot\mathbf{p}$ theory [6] as

$$d_k^{\lambda\lambda'} = d_0^{\lambda\lambda'}\frac{E_g^{\lambda\lambda'}}{\epsilon_k^\lambda - \epsilon_k^{\lambda'}}, \tag{8}$$

where $d_0^{\lambda\lambda'}$ resembles the transition dipole moment between bands λ and λ' at the Γ-point, and $E_g^{\lambda\lambda'}$ the bandgap energy at the Γ-point between bands λ and λ'. The semiconductor Bloch equations are cast as a set of coupled partial differential equations with periodic boundaries. To solve this numerical problem, the k-dimension of the equations are represented in a Fourier-series base and then solved by using spectral methods, as provided through the Dedalus project [7].
The macroscopic polarization $P(t)$ and macroscopic current $J(t)$ are calculated [4] by

$$P(t) = \sum_{\lambda,\lambda',k}[d_k^{\lambda\lambda'}p_k^{\lambda\lambda'} + c.c.] \tag{9}$$

$$J(t) = \sum_{\lambda,k} v^\lambda(k)f_k^\lambda \tag{10}$$

with the group velocity $v^\lambda(k)$ given by

$$v^\lambda(k) = \nabla_k\epsilon_k^\lambda. \tag{11}$$

We calculate the spectral representation of the source field of interband polarization and intraband current as follows

$$F_{\mathrm{inter}}(\omega) = \mathcal{F}[\frac{\partial}{\partial t}j_{\mathrm{inter}}(t)] = \mathcal{F}[\frac{\partial^2}{\partial^2 t}P(t)] = \omega^2\mathcal{F}[P(t)] = \omega^2 P(\omega). \tag{12}$$

and the current as

$$F_{\mathrm{intra}}(\omega) = \mathcal{F}[\frac{\partial}{\partial t}j_{\mathrm{intra}}(t)] = i\omega\mathcal{F}[J(t)] = i\omega J(\omega). \tag{13}$$

The harmonic spectral density at the sample plane is then defined as

$$I_{\mathrm{HHG}}(\omega) = |\omega^2 P(\omega) + i\omega J(\omega)|^2. \tag{14}$$

B. Far-field propagation

The SBE yield the spatial distribution of the complex electric field for the interband and intraband contributions $F_{\text{inter}}(\omega)$ and $F_{\text{intra}}(\omega)$, respectively, at the sample plane. Detection is performed in the far-field which, in the Fraunhofer diffraction regime, is related to the sample plane through a Fourier transform:

$$\mathcal{F}[F_{\text{HHG}}(x_{SP},\omega)] \propto F_{\text{HHG}}(x_{FF},\omega). \tag{15}$$

The field at the sample plane is described by a one-dimensional spatial variable x_{SP}. To obtain a far-field pattern, the harmonic signal $F_{\text{HHG}} = F_{\text{inter}} + F_{\text{intra}}$ is computed for every value of x_{SP}. The near-field spectrum of the solid HHG is propagated to the far-field by Fraunhofer diffraction, which gives the wave mixing pattern shown in Fig. 2(b) in the main text. The total field is built up by an 800-nm pulse and a 400-nm pulse, which are crossed under an angle β, thus creating a time-dependent grating-like interference pattern. The FWHM size of the focus of the 800-nm pulse is measured to be 130 µm, the 400-nm focus is measured to be 100 µm. In the focal plane, both pulses are assumed to have a flat spatial phase front. The only remaining phase factor is introduced by the angle β between the two pulses, which depends on the position x, $\phi(x) \approx x\sin(\beta)$.

C. Separation into band-resolved interband-polarization and intraband-current contributions

The total harmonic signal in the wavemixing configuration of Fig. 2b (main text) is calculated by summing up all contributions along the sample plane, see Fig. S1a. To identify which bands contribute most to the signal, we calculate the separate values of the interband polarization (Fig. S1b) and the intraband current (Fig. S1c)). Overall, the interband polarization dominates the emission in the spectral region around the bandgap (H5-H11). The main contribution of the intraband current originates from the carriers inside the first conduction band. The main contribution of the interband polarization is generated between the valence and the lowest lying conduction band.

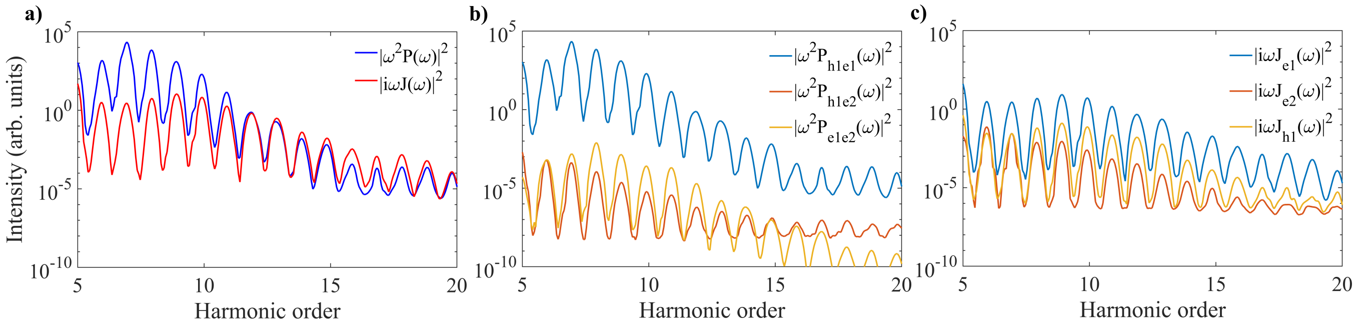

Figure S1: a) Total interband polarization and intraband current. b) Separate contributions of the interband polarization. c) Separate contributions of the intraband current.

D. Electronic-structure calculations for material parameters of silica

The energy levels and the transition dipole moments of fused silica at the Γ-point between the valence band and two conduction bands are calculated within density functional theory, using pseudopotentials (generalised gradient approximation in the shape of the Perdew, Burke, and Ernzerhof (PBE) functional [8]) and a plane-wave basis set as implemented in Quantum Espresso [9]. The plane-wave energy cutoff used was 40 Ry. The calculation is performed for an automatically generated uniform grid of 10 points in each k direction. Both these parameters were tested for convergence. The energy levels at k = 0 are calculated to be 0 eV, 9.6 eV and 12.6 for the valence and the two lowest lying conduction bands respectively. The dipole moments between these energy levels λ, λ' are calculated to be $d_0^{h_1e_1} = 0.318$ a.u., $d_0^{e_1e_2} = 0.014$ a.u. and $d_0^{e_2h_1} = 0.037$ a.u., using the epsilon function from the post processing data package. The dispersion relation of the bands of silica are taken from [10]. We model the light-matter interaction along the Γ-M crystal direction of quartz, as this orientation generates the most harmonic emission [10].

E. Semiclassical description of laser-driven carrier motion

The semiclassical description [10, 11] of the carrier dynamics inside an energy band λ starts with describing the velocity of an electronic wavepacket by

$$\mathbf{v}^{\lambda}(\mathbf{k},t) = \frac{\partial \epsilon^{\lambda}(\mathbf{k})}{\partial \mathbf{k}}. \tag{16}$$

The crystal momentum is time-dependent, given by

$$\mathbf{K}(t) = \mathbf{k} + \mathbf{A}(t), \tag{17}$$

with the vector potential,

$$\mathbf{A}(t) = -\int_{\infty}^{t} \mathbf{F}(t')dt' \tag{18}$$

based on the two-color laser field $\mathbf{F}(t) = F_{800}\cos(\omega t) + F_{400}\cos(2\omega t + \phi)$, with the field amplitudes of the fundamental (F_{800}) and second harmonic (F_{400}) field, and the relative phase ϕ between the fields. By expressing the dispersion relation of band λ as,

$$\epsilon^{\lambda}(\mathbf{k}) = \sum_{\mathbf{r}} E^{\lambda}(\mathbf{r})\cos(\mathbf{k}\cdot\mathbf{r}) \tag{19}$$

in which the band coefficients $E^{\lambda}(\mathbf{r})$ represent the amplitude of the spatial harmonics of the lattice structure, and by substituting the time-dependent crystal momentum we obtain a final expression for the velocity

$$\mathbf{v}^{\lambda}(\mathbf{k},t) = -\sum_{\mathbf{r}} \mathbf{r}E^{\lambda}(\mathbf{r})\sin[(\mathbf{k}+\mathbf{A}(t))\cdot\mathbf{r}]. \tag{20}$$

We calculate the harmonic intensity as

$$I_{\mathrm{HHG}}(\omega) \propto \sum_{\mathbf{k}} |\omega\mathcal{F}[\mathbf{v}^{\lambda}(\mathbf{k},t)]|^2. \tag{21}$$

To reproduce the intensity scalings, we calculate the spectrum for each combination of intensities of the fundamental and second harmonic in Fig. 3 of the main text. The semiconductor Bloch simulations reveal that the main contribution is coming from the polarization between the valence band and the first conduction band. In addition, the nonlinearity of the valence band is much lower than for the first conduction band, such that the main emission will originate from the first conduction band. Therefore we calculate the spectrum used in the intensity scalings only based on the dispersion relation of the first conduction band.

III. ADDITIONAL EXPERIMENTAL DATA AND ANALYSIS

A. Comparison of WMOs generated with orthogonal or parallel polarizations of the 400-nm and 800-nm pulses

Fig. S2 shows the far-field XUV wave mixing emission signals in silica recorded for 400-nm and 800-nm pulses with orthogonal (Fig. S2 (a)) and parallel (Fig. S2 (b)) polarization orientations of both pulses under identical experimental conditions. The experiments were performed for the constant intensity of the 400-nm and 800-nm pulses of 1.6 TW/cm^2 and 12 TW/cm^2, respectively. The polarization of the two beams was selected by rotating two separate half-wave plates, which were positioned in both arms. As depicted in Fig. S2 (c), when comparing the two polarization scenarios, it is evident that the yield of WMOs is significantly higher for parallel polarizations than for the case of orthogonal polarizations. In particular, the results demonstrate that for parallel polarizations, the emission signal from wave mixing for harmonic 7, harmonic 9, and harmonic 10 is more than 6 times greater than for orthogonally polarised pulses. Additionally, several of the WMOs, such as (5,2), (4,3), (8,1), and (7,2), which were hardly visible for the orthogonal case, are distinctly apparent for the parallel two-pulse polarization alignment geometry.

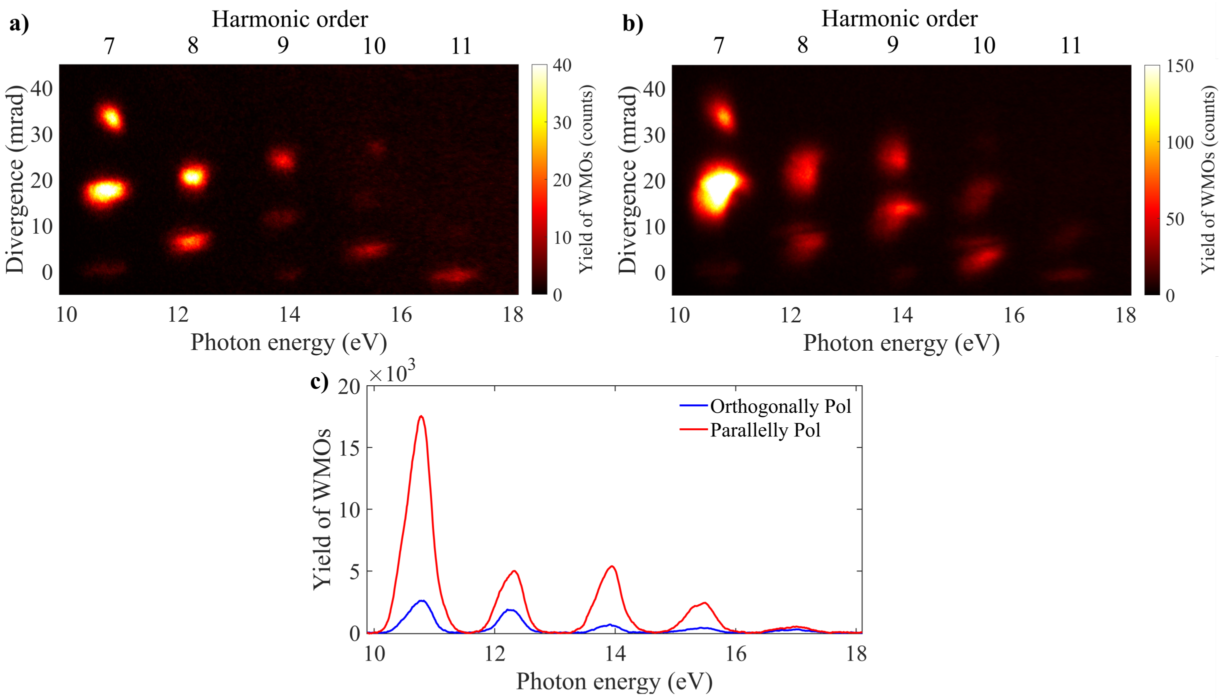

Figure S2: Far-field XUV wave mixing spectra in silica recorded for a) orthogonal polarizations and b) parallel polarizations for 400-nm and 800-nm pulses. c) Comparison of the photon-energy-integrated yield of WMOs generated with orthogonal or parallel polarizations of the 400-nm and 800-nm pulses. The intensity of the 400-nm and 800-nm pulses was 1.6 TW/cm^2 and 12 TW/cm^2, respectively.

B. Yield of harmonics generated by 800-nm and 400-nm-pulses-only in comparison with wave mixing

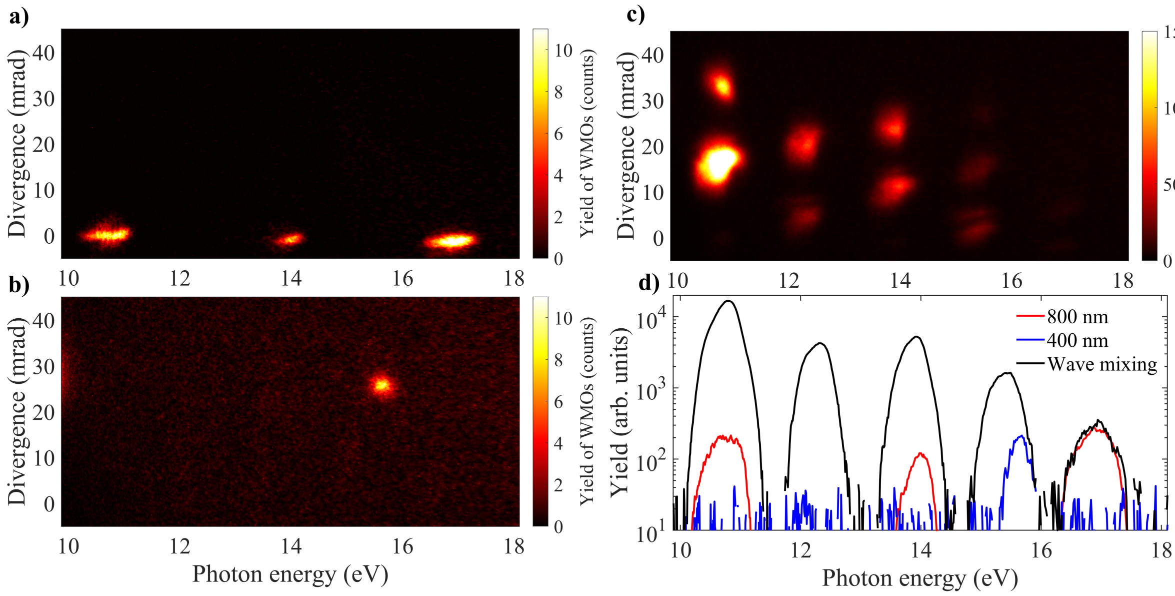

Figure S3: Far-field XUV spectra in silica recorded with a) 800-nm, b) 400-nm-only pulses, and c) high-order wave mixing with the 800-nm and 400-nm pulses combined. The total intensity of 13.6 TW/cm^2 for both colours combined was equal to the intensity of a single 800-nm pulse. d) The corresponding spectrally-resolved and divergence-integrated yields of XUV emission for single-colour HHG or wave mixing, on a logarithmic y-scale.

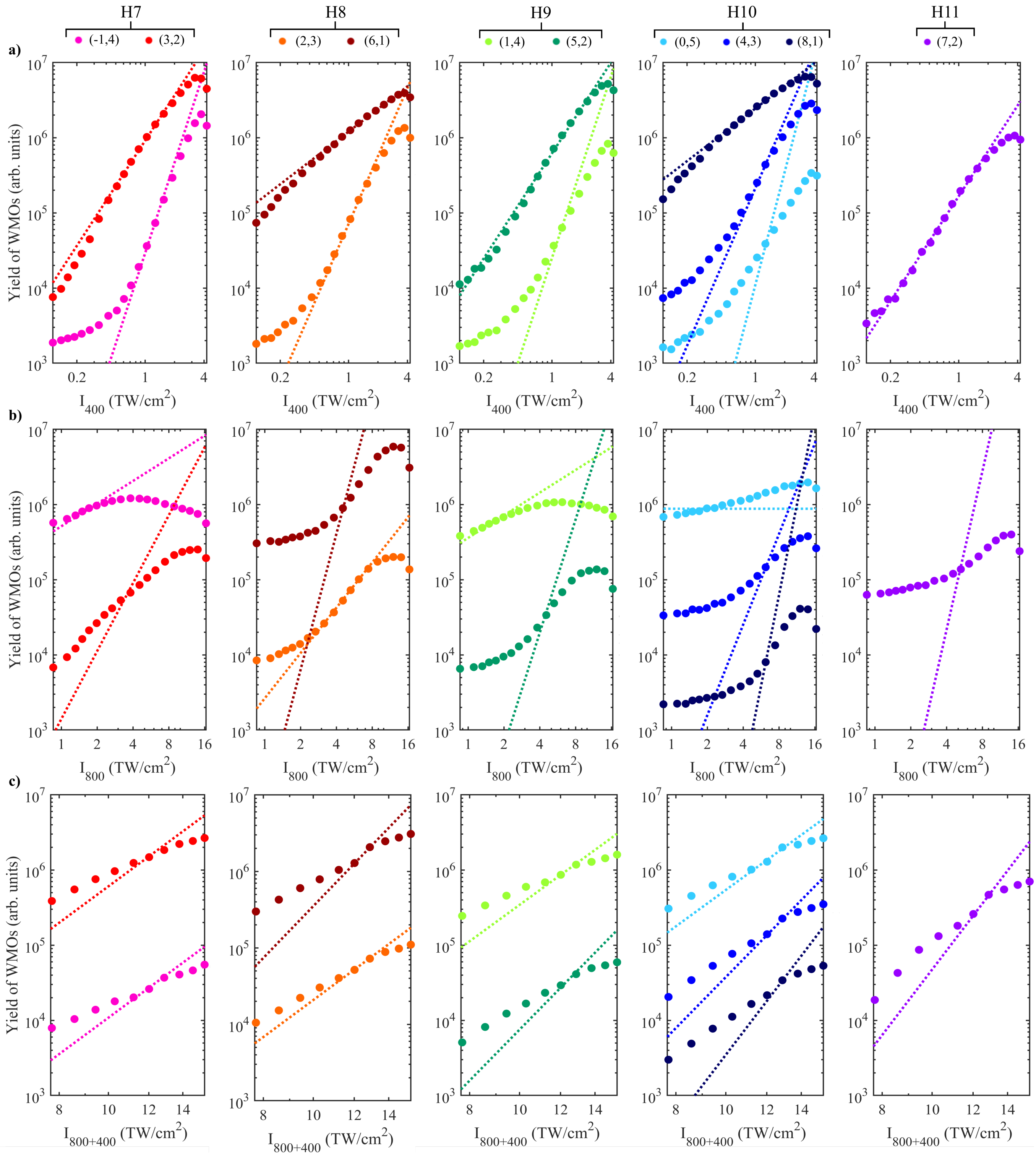

Figure S4: Comparison between experimental data (circles) and semi-classical simulations (dashed lines) of different WMOs. a) Only the intensity of the 400-nm pulse is scaled (I_{800}=10.5 TW/cm^2). b) Only the intensity of the 800-nm pulse is scaled (I_{400}=1.6 TW/cm^2). c) The total intensity of two colors is scaled (the ratio I_{800}/I_{400} is 3.3:1).

The WMOs generated from silica driven by 800-nm and 400-nm pulses show higher signals in all experiments than HHG generated with either 800-nm or 400-nm pulses alone. Fig. S3 shows the far-field XUV wave mixing spectra in silica recorded with 800-nm (a), 400-nm-only (b) pulses, and (c) wave mixing signals of both pulses combined. All scans were taken under identical experimental conditions. The total intensity of 13.6 TW/cm^2 (1.6 TW/cm^2 at 400-nm and 12 TW/cm^2 at 800-nm, respectively) was used to generate the WM signal using two pulses, which was matched to the intensity of a single 800-nm colour at 13.6 TW/cm^2 in the HHG experiments driven by the 800-nm pulse alone. A comparison was made between the yield of WMOs generated by both 800-nm and 400-nm pulses and the yield of on-axis HHG produced by 800-nm and 400-nm only at the same photon energy to determine the XUV wave mixing yield, as shown in panel (c) of Fig. S3. Importantly, the yield of harmonic order (7,0) at 10.8 eV is 78 times greater in the case of wave mixing compared to the on-axis harmonic (7,0) driven solely by an 800-nm pulse. An yield enhancement factor of about 43 is found for the signal of WMO (9,0). Even though the yield enhancement is slight (factor of 1.2) for WMO (11,0), the signal of WMO (7,0) is 68 times larger than the signal of the on-axis harmonic (11,0). Additional measurements were performed to support these findings and calculate the WH harmonic photon flux, giving insights into the wave mixing conversion efficiency in SiO_2, as detailed in the following section. Fig. S4 shows all measured WMOs, a selection of those is shown in Fig. 3 in the main text. The measured data is displayed as circles and the simulated data, based on the semiclassical description of laser-driven carrier motion (see section II.E), as dashed lines.

IV. WAVE MIXING CONVERSION EFFICIENCY IN SILICA

A. Experimental details

To verify the conversion efficiency of high-order wave mixing in silica (SiO_2), separate measurements implementing photon flux detection were performed. Due to the low amount of XUV photons generated by SiO_2 and a relatively high background signal, photon flux from SiO_2 could not be directly detected using solely an XUV photodiode. Nevertheless, the photon flux was quantified by comparing the spectrally resolved far-field XUV emission patterns from SiO_2 to that of xenon (Xe) gas. The absolute HHG flux of wave mixing from Xe was measured with a calibrated photodiode to provide an absolute standard for the photon flux. Xe gas, which has an ionization potential of 12.13 eV [12], was deliberately selected owing to its capacity to produce lower harmonic photon energies that align with those of SiO_2, allowing for high enough XUV photon counts to be detected with the photodiode used in the experiments. A schematic layout of the photon flux detection setup and propagation of the 800 nm, 400 nm, and XUV beams in the configuration used in the Xe gas and SiO_2 wave mixing experiments is shown in Fig. S5.

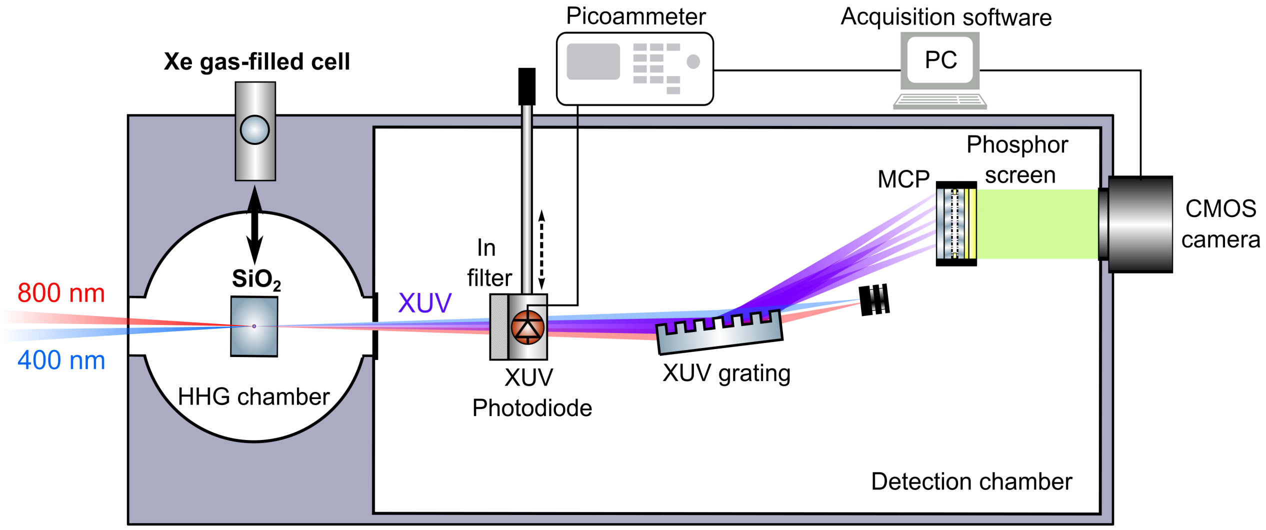

Figure S5: Schematic layout of the photon flux detection setup and propagation of the 800 nm, 400 nm, and XUV beams in the configuration used in the gas and solid wave mixing experiments.

First, the fundamental (800 nm) and second harmonic (400 nm) beams were focused non-collinearly in a gas cell filled with Xe, generating low-order harmonics 7 – 13 of the fundamental frequency. Thereafter, the two remaining driving beams that were still propagating along their harmonic beam paths were filtered out by a 154 nm thick indium (In) foil spectral filter, supported by a nickel mesh. The spectral response of the filter is shown by the blue curve in Fig. S7(a). The remaining XUV wave mixing signal was detected by an XUV photodiode (AXUV100G, OptoDiode), which was individually calibrated by the Physikalisch-Technische Bundesantalt (PTB). The photodiode had a large active area of 10 x 10 mm and a relatively high responsivity in the XUV-UV spectral range, as shown by the orange curve in Fig. S7(b). Consequently, the photocurrent of the photodiode was read out with the use of a picoammeter (Model 6485, Keithley). It is important to note that only harmonic orders 8, 9, and 10 could be recorded by the photodiode due to the limited spectral transmission range of the In filter. Then, the photodiode and the filter were moved out of the beam path, using a linear feedthrough, allowing the harmonics to be spectrally dispersed and collected by a double-stack microchannel plate (MCP) detector combined with a phosphor screen (Photonis Inc.), which were then imaged by a CMOS camera. The SiO_2 sample was mounted below the gas cell on the same high-precision manipulator assembly, enabling easy transition between Xe and SiO_2 by raising the gas cell and moving the SiO_2 into focus. It is important to emphasize that in the SiO_2 sample, wave mixing was achieved at laser pulse peak intensities at the focus in the order of several TW/cm^2 due to the lower damage threshold of solids. In comparison, Xe gas required intensities

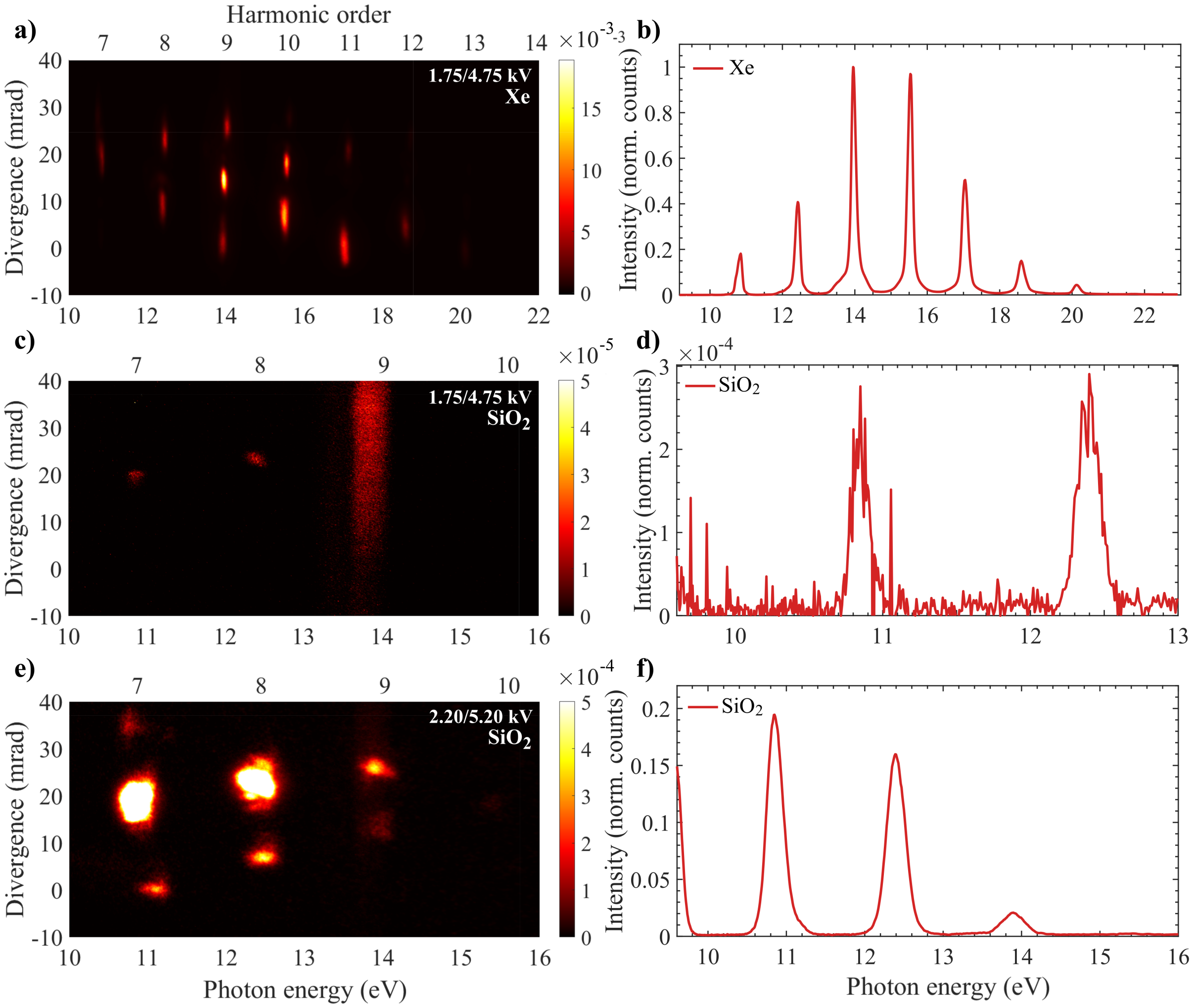

Figure S6: The far-field XUV wave mixing emission signals recorded from Xe gas and silica (left panels) and the photon-energy-integrated harmonic intensity signals (right panels). The upper and middle panels represent the harmonic signal obtained from Xe (a, b) and SiO_2 (c, d), respectively, using the same MCP/phosphor screen voltages of 1.75/4.75 kV. The lower panels show the harmonic signal from SiO_2, recorded at higher MCP/phosphor screen voltages of 2.20/5.20 kV. The spectra displayed in the right panels are normalized to the maximum Xe signal (b). The color bar indicates the relative magnitude of the harmonic yield.

of $\geq 10^{13}$ W/cm^2. Consequently, the harmonic wave mixing emission signals are also lower in SiO_2, impeding direct measurement of the corresponding photon flux using a photodiode. Therefore, the MCP signal of SiO_2 was calibrated using the photon flux measurement of Xe.

B. Results

Fig. S6 shows the far-field XUV wave mixing emission signals from Xe (a) and SiO_2 (c) under the same acquisition conditions with MCP/phosphor screen voltages of 1.75/4.75 kV, and (e) shows the far-field emission for SiO_2 with a higher MCP/phosphor screen voltage of 2.20/5.20 kV and otherwise same acquisition conditions. The right panels Fig. S6(b,d,f) show the corresponding divergence-integrated intensity signals. The spectra shown in Fig. S6 have been normalized to the maximum Xe signal, which is displayed in Fig. S6(b). The calibration of the horizontal axes of all harmonic spectra from wavelength to energy was done by multiplying the data by the Jacobian transformation [14]. The photon flux calibration for SiO_2 is done as follows. First the photon flux per harmonic order of Xe is calibrated. The total XUV power after the Indium filter is calculated by,

$$P_{tot} = \frac{I_{PD}}{\sum_{q=8}^{11}(r_q\, S_q)}. \tag{22}$$

Where I_{PD} is the measured photon current, r_q is the ratio of harmonic peak q to the sum of the transmitted harmonic peaks ($\sum_{q=8}^{11} r_q = 1$) and S_q is the photodiode spectral responsivity per harmonic order, see Fig. S7(b). Then the generated XUV pulse energy at source and per harmonic order is given by,

$$E_q = \frac{r_q\, P_{tot}}{f_{rep}\, T_q}. \tag{23}$$

Here, T_q is the transmission of the Indium filter, see Fig. S7(a) and $f_{rep} = 1$ kHz is the repetition rate of the laser. Due to the nonlinear response of the MCP to photon flux (as well as the applied MCP voltage) and a three order

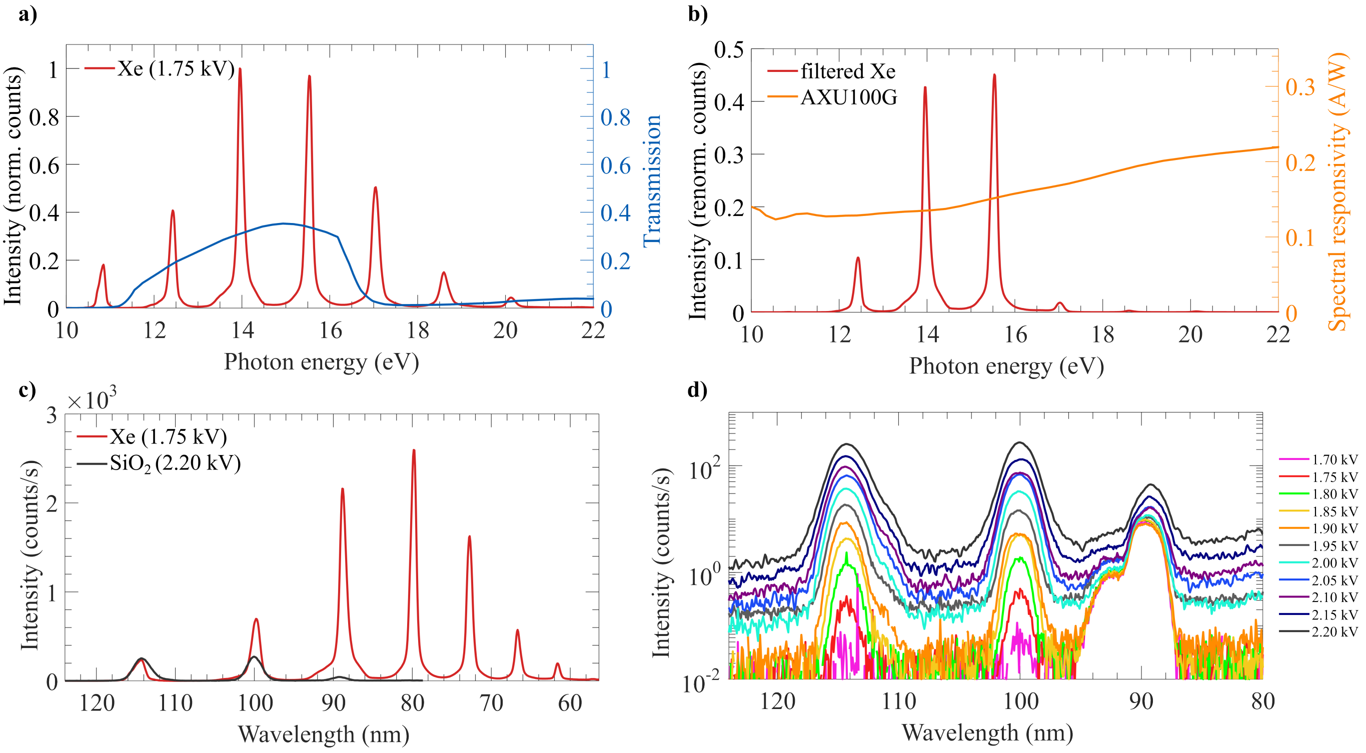

Figure S7: a) The spectrum of the wave mixing emission signal recorded in Xe gas and the transmission function of a 154 nm thick indium XUV spectral filter (data taken from Ref. [13]). b) The transmitted spectrum from Xe and calibrated spectral responsivity of the AXUV100G photodiode, as provided by the PTB. c) The spectra, in wavelength, of the SiO_2 in black and Xe in red, at 1.75 kV and 2.20 kV MCP voltage respectively. d) The spectra of the wave mixing emission signal recorded from SiO_2 at varying MCP voltages.

of magnitude difference between the SiO_2 and Xe spectral amplitude at equal MCP voltage, taking the ratio at this equal MCP voltage would underestimate the SiO_2 XUV photon flux due to the well-known nonlinearity of the MCP response as a function of actual incident photon flux. This underestimation is avoided when comparing MCP signal of SiO_2 (2.20 kV) and Xe (1.75 kV), which are at the same order of magnitude, see Fig. S7(c), and correcting for the measured gain factor of the MCP between 1.75 kV and 2.20 kV that was independently measured for the SiO_2 signal. The final XUV pulse energy for harmonic 8 of SiO_2 is then calibrated to be,

$$E_{H8}^{\mathrm{SiO_2}} = \frac{R}{G} E_{H8}^{\mathrm{Xe}}. \tag{24}$$

The ratio between harmonic 8 of SiO_2 (2.20 kV) and Xe (1.75 kV) is given by $R = \int_{95}^{105} A_{\mathrm{SiO_2}} d\lambda(\mathrm{nm}) / \int_{95}^{105} A_{\mathrm{Xe}} d\lambda(\mathrm{nm})$ and the gain factor is taken as the ratio of the sum of counts of the SiO_2 spectrum at 2.20 kV and 1.75 kV $G = \sum A_{\mathrm{SiO_2}}(2.20\ \mathrm{kV}) / \sum A_{\mathrm{SiO_2}}(1.75\ \mathrm{kV})$, see Fig. S7(d). Lastly, the XUV pulse energy from harmonic 7 and 9 of SiO_2 are calculated using their integrated relative ratio's to harmonic 8 at 2.20 kV. The results of the XUV pulse energy measurements are summarized in table I. The total CE of WM in SiO_2 is determined to be $5.60 \pm 1.16 \cdot 10^{-10}$. As described in Section III, the efficiency of 800-nm single-color solid HHG in silica within the $10 - 13$ eV photon energy range was observed to be one to two orders of magnitude lower, depending on the specific harmonic order. This places the NIR single-color SiO_2 CE in the order of magnitude of 10^{-11} - 10^{-12}. The observed Xe CE is lower than what is typically expected; however, the generation conditions were intentionally not optimized to still be able to detect the signal at the same MCP voltage for SiO_2, without saturating the Xe. Fig. S8 shows the measured photocurrent as a function of backing pressure. It is clear that the backing pressure is not saturated.

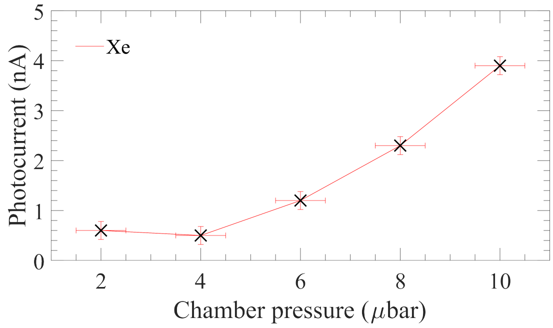

Figure S8: Photocurrent as measured from the XUV photodiode at varying Xe gas cell pressures. The latter was not directly measured. The horizontal axis is the measured pressure in the HHG chamber.

HHG medium	**SiO_2**	**Xe**	**Ar**
Driver (nm)	800 + 400	800 + 400	Single-color 800
Input pulse energy (J)	$9.3 \cdot 10^{-5}$	$6.7 \cdot 10^{-4}$	$1.40{\cdot}10^{-3}$
Chamber gas pressure (bar)	–	low ($2 \cdot 10^{-6}$)	high ($7 \cdot 10^{-3}$)
Photon energy range (eV)	10 - 13	10 - 21	20 - 45
Spectral filter (eV)	In (11 - 17)	In (11 - 17)	Al (15 - 73)
Measured photocurrent (nA)	–	0.6 ± 0.18	171
XUV photon flux (photons/s)			
Harmonic 7	$1.42 \pm 0.45 \cdot 10^7$	$0.53 \pm 0.17 \cdot 10^9$	
Harmonic 8	$1.15 \pm 0.37 \cdot 10^7$	$1.19 \pm 0.38 \cdot 10^9$	
Harmonic 9	$0.20 \pm 0.06 \cdot 10^7$	$2.60 \pm 0.83 \cdot 10^9$	
Harmonic 10	–	$2.27 \pm 0.72 \cdot 10^9$	
Harmonic 11	–	$1.07 \pm 0.34 \cdot 10^9$	
Harmonic 12	–	$0.40 \pm 0.13 \cdot 10^9$	
Harmonic 13	–	$0.12 \pm 0.04 \cdot 10^9$	
Total	$2.77 \pm 0.59 \cdot 10^7$	$8.17 \pm 1.23 \cdot 10^9$	$2.35 \cdot 10^{11}$
Conversion efficiency, CE	$5.60 \pm 1.16 \cdot 10^{-10}$	$2.87 \pm 0.43 \cdot 10^{-8}$	$8.15 \cdot 10^{-7}$

TABLE I: The XUV generation conditions and the extracted parameters obtained from the wave mixing measurements in SiO_2 solid and Xe gas in comparison to the regular single-color (800 nm) measurements in Ar gas.

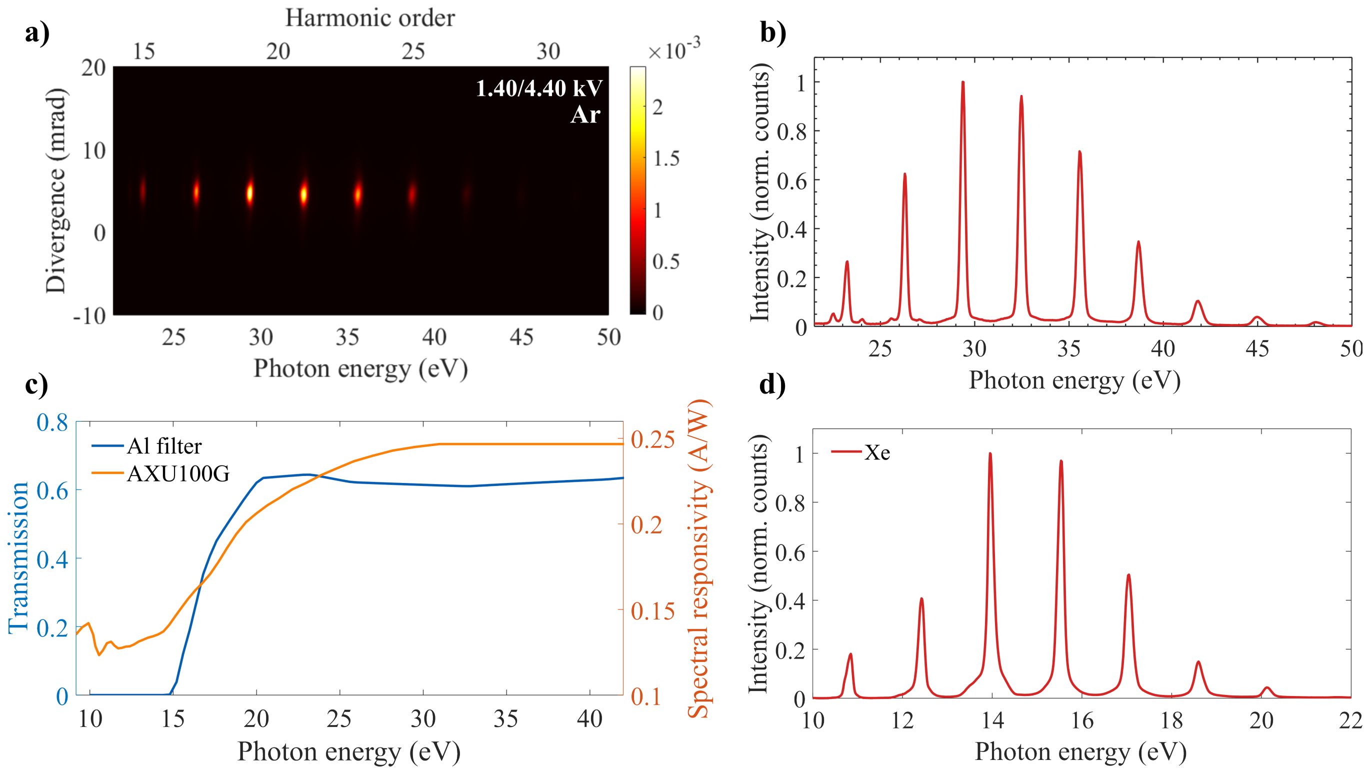

Figure S9: a) The recorded far-field XUV emission signal from Ar gas. The color bar indicates the relative magnitude of the harmonic yield. b) The normalized to the maximum intensity signal harmonic spectrum of Ar gas as a function of photon energy. c) The transmission curve of a 200 nm thick aluminium spectral filter (blue) and the spectral responsivity of the XUV photodiode (yellow). d) The XUV wave mixing emission signal recorded in Xe gas, as presented in Fig. S6 (b).

To verify the reliability of the photon flux measurements of Xe, the Xe data was compared to a more optimized XUV photon flux measurements of argon (Ar) gas. Given Ar's higher ionization potential (15.6 eV) and ionization saturation intensity, the harmonic range observed in gas-phase experiments is wider for Ar (20 - 50 eV) compared to Xe (10 - 21 eV). Therefore, a 200 nm thick aluminium (Al) spectral filter was utilized instead of the previous 154 nm thick indium filter to effectively eliminate the fundamental light and isolate the generated harmonics from Ar. Additionally, the gas cell was sealed with a kapton foil that was pasted around it, which allowed a small hole through the wrapping to be drilled by the focused laser beam, increasing the effective gas pressure in the cell. Lastly, instead of the two-color (fundamental and second harmonic) non-collinear configuration, only the fundamental 800 nm beam was used due to the necessity of comparing the measured XUV photon energy to previous literature findings under similar conditions. The rest of the experimental configuration remained consistent with the details provided in Fig. S5. The single-color XUV harmonic spectra of Ar are shown in Fig. S9(a) and (b), where the former is the far-field image of the XUV emission signal on the MCP as recorded by the CMOS camera and the latter is a projection on the photon energy axis and normalized to the maximum Ar signal. Fig. S9(c) shows the spectral transmission of the Al filter and the XUV photodiode responsivity for the energy range associated with the harmonics produced using Ar. The sensitivity of the photodiode is considered to remain constant above 30 eV due to the restricted calibration range provided by PTB. Table I summarizes the generation conditions, data acquisition settings, and XUV photon flux results for SiO_2, Xe and Ar. In Ar gas, the measured CE reached $8.15 \cdot 10^{-7}$. This is consistent with typical single-color HHG values in Ar that typically mention conversion efficiencies around $\cdot 10^{-6}$. As mentioned above, the conditions in Xe were on purpose detuned to make the fluxes in solid and gas XUV generation more comparable.

[1] J. Bertrand, H. J. Wörner, H.-C. Bandulet, É. Bisson, M. Spanner, J.-C. Kieffer, D. Villeneuve, and P. B. Corkum, Phys. Rev. Lett **106**, 023001 (2011).

[2] C. M. Heyl, P. Rudawski, F. Brizuela, S. N. Bengtsson, J. Mauritsson, and A. L'Huillier, Phys. Rev. Lett. **112**, 1 (2014).
[3] M. Lindberg and S. W. Koch, Phys. Rev. B **38**, 3342 (1988).
[4] D. Golde, T. Meier, and S. W. Koch, Phys. Rev. B **77**, 075330 (2008).
[5] O. Schubert, M. Hohenleutner, F. Langer, B. Urbanek, C. Lange, U. Huttner, D. Golde, T. Meier, M. Kira, S. W. Koch, et al., Nat. Photonics **8**, 119 (2014).
[6] H. Haug and S. W. Koch, *Quantum theory of the optical and electronic properties of semiconductors* (World Scientific Publishing Company, 2009).
[7] K. J. Burns, G. M. Vasil, J. S. Oishi, D. Lecoanet, and B. P. Brown, Phys. Rev. Res. **2**, 023068 (2020).
[8] J. P. Perdew, K. Burke, and M. Ernzerhof, Physical review letters **77**, 3865 (1996).
[9] P. Giannozzi, O. Andreussi, T. Brumme, O. Bunau, M. B. Nardelli, M. Calandra, R. Car, C. Cavazzoni, D. Ceresoli, M. Cococcioni, et al., J. Condens. Matter Phys **29**, 465901 (2017), URL `http://stacks.iop.org/0953-8984/29/i=46/a=465901`.
[10] T. T. Luu, M. Garg, S. Y. Kruchinin, A. Moulet, M. T. Hassan, and E. Goulielmakis, Nature **521**, 498 (2015).
[11] M. Wegener, *Extreme nonlinear optics: an introduction* (Springer Science & Business Media, 2005).
[12] A. Kramida, Y. Ralchenko, J. Reader, and N. A. Team, *NIST Atomic Spectra Database* (National Institute of Standards and Technology, Gaithersburg, 2020).
[13] B. Henke, E. Gullikson, and J. Davis, Atomic Data and Nuclear Data Tables **54**, 181 (1993).
[14] J. Mooney and P. Kambhampati, J. Phys. Chem. Lett. **4**, 3316–3318 (2013).
